# A prognostic model based on DNA methylation-related gene expression for predicting overall survival in hepatocellular carcinoma

**DOI:** 10.3389/fonc.2023.1171932

**Published:** 2024-01-18

**Authors:** Jin Luo, Wan-Cui Zhu, Qiu-Xia Chen, Chang-Fu Yang, Bi-Jun Huang, Shi-Jun Zhang

**Affiliations:** ^1^ Department of Traditional Chinese Medicine, The First Affiliated Hospital, Sun Yat-sen University, Guangzhou, China; ^2^ Department of Traditional Chinese Medicine, Shenzhen Children’s Hospital, Shenzhen, Guangdong, China; ^3^ State Key Laboratory of Oncology in South China, Guangdong Key Laboratory of Nasopharyngeal Carcinoma Diagnosis and Therapy, Sun Yat-sen University Cancer Center, Collaborative Innovation Center for Cancer Medicine, Guangzhou, China; ^4^ Department of Oncology, The People’s Hospital of Gaozhou, Gaozhou, China; ^5^ Department of Experimental Research, State Key Laboratory of Oncology in South China, Collaborative Innovation Center for Cancer Medicine, Sun Yat-Sen University Cancer Center, Guangzhou, China

**Keywords:** prognosis, prognostic survival model, DNA methylation, risk score, hepatocellular carcinoma

## Abstract

**Background:**

Hepatocellular carcinoma (HCC) continues to increase in morbidity and mortality among all types of cancer. DNA methylation, an important epigenetic modification, is associated with cancer occurrence and progression. The objective of this study was to establish a model based on DNA methylation risk scores for identifying new potential therapeutic targets in HCC and preventing cancer progression.

**Methods:**

Transcriptomic, clinical, and DNA methylation data on 374 tumor tissues and 50 adjacent normal tissues were downloaded from The Cancer Genome Atlas–Liver Hepatocellular Carcinoma database. The gene expression profiles of the GSE54236 liver cancer dataset, which contains data on 161 liver tissue samples, were obtained from the Gene Expression Omnibus database. We analyzed the relationship between DNA methylation and gene expression levels after identifying the differentially methylated and expressed genes. Then, we developed and validated a risk score model based on the DNA methylation-driven genes. A tissue array consisting of 30 human hepatocellular carcinoma samples and adjacent normal tissues was used to assess the protein and mRNA expression levels of the marker genes by immunohistochemistry and qRT-PCR, respectively.

**Results:**

Three methylation-related differential genes were identified in our study: GLS, MEX3B, and GNA14. The results revealed that their DNA methylation levels were negatively correlated with local gene expression regulation. The gene methylation levels correlated strongly with the prognosis of patients with liver cancer. This was confirmed by qRT-PCR and immunohistochemical verification of the expression of these genes or proteins in tumors and adjacent tissues. These results revealed the relationship between the level of relevant gene methylation and the prognosis of patients with liver cancer as well as the underlying cellular and biological mechanisms. This allows our gene signature to provide more accurate and appropriate predictions for clinical applications.

**Conclusion:**

Through bioinformatics analysis and experimental validation, we obtained three DNA methylation marker: GLS, MEX3B, and GNA14. This helps to predict the prognosis and may be a potential therapeutic target for HCC patients.

## Background

Despite that hepatocellular carcinoma (HCC) ranks fifth and second among the various types of cancer in terms of incidence and mortality rate, respectively (1–3), its etiology and pathogenesis remain unclear. Liver transplantation is the treatment of choice for early-stage HCC, whereas surgical resection, radiofrequency ablation and transarterial chemoembolization, broad-spectrum tyrosine kinase inhibitors (e.g., sorafenib and levatinib), and a combination of immunotherapy and antiangiogenic therapy (e.g., azelizumab) are the mainstay of treatment for patients with advanced HCC. However, these anticancer agents provide only a nominal extension in survival and generate a wide range of severe side effects, and patients eventually acquire drug resistance. Some common mutations in HCC-related genes, such as those encoding telomerase reverse transcriptase (TERT), tumor protein p53 (TP53), and catenin beta 1 (CTNNB1), are considered to be irreversible. Both the identification of relevant gene targets and selective gene delivery are important in this scenario (4, 5). Therefore, the development of molecular biomarkers for predicting prognosis and treatment outcomes in HCC is crucial.

As one of the best studied of the various epigenetic modifications, DNA methylation ensures the regulation of correct gene expression and stable gene silencing in normal mammalian cells. DNA methylation is linked to histone alterations, where the interaction between these two epigenetic modifications for altering the chromatin architecture is critical for regulating genome function. DNA methyltransferases are the enzymes responsible for the development and maintenance of methylation patterns.

It is well known that hypermethylation within promoter regions causes the inactivation of several tumor suppressor genes. Numerous studies have identified a wide range of genes that are silenced by DNA methylation in various cancer types. By contrast, global hypomethylation, which causes genomic instability, mediates cell transformation. In addition to the changes it generates in promoter regions and repetitive DNA sequences, DNA methylation is linked to the regulation of the production of noncoding RNAs, such as microRNAs, which may also play a role in tumor suppression. DNA methylation appears to be promising in terms of its potential translational applications in the clinical setting, such as the use of hypermethylated promoters as biomarkers of diseases. Furthermore, unlike hereditary changes, DNA methylation is reversible, making it a particularly appealing therapeutic strategy. The significance of changes in DNA methylation in cancer has motivated us to decode the human epigenome, albeit different DNA methylome mapping approaches are still required to complete this endeavor (6, 7).

Methylation can occur at both the DNA and protein levels. As a covalent chemical modification process, DNA methylation is critical for many biological processes (8), including genomic imprinting (9), gene expression regulation (10), cell differentiation (11), development (12), and inflammation (13). Aberrant DNA methylation has been linked to various diseases, including cancer (14, 15). In normal cells, CpG dinucleotides are grouped into large clusters known as CpG islands and most are unmethylated. One of the most commonly observed alterations in tumor cells is the hypermethylation of cytosine residues within CpGs, which may result in the silencing of tumor suppressor genes (16). For example, hypermethylation has been found in the breast cancer gene 1 (*BRCA1*) and target of methylation-induced silencing 1 (*TMS1*) gene promoters in breast cancer cells (17), the death-associated protein kinase (*DAPK*), Ras association domain family member 1 (*RASSF1A*) gene promoters in lung cancer cells (18) and the cyclin-dependent kinase inhibitor 2A (*CDKN2A*) gene promoter in colon cancer cells (19). Several studies (20, 21) have found that DNA methylation markers are more sensitive than protein markers for diagnostic purposes, suggesting that cancer-specific DNA methylation markers have a high potential for use in clinical diagnosis.

In this study, we screened out 17 DNA methylation-driven genes from the Cancer Genome Atlas data portal (TCGA; http://cancergenome.nih.gov/) and Gene Expression Omnibus (GEO) database. A prognostic survival model based on DNA methylation-related gene expression in HCC was established using the thresholds of an absolute log fold-change (FC) value of greater than 1 and a *p* value of less than 0.05. Finally, genes whose expression levels were negatively correlated with their methylation levels were identified as DNA methylation-driven genes. The prognostic survival model could provide insightful suggestions for exploring the mechanisms of HCC development and progression as well as treatment strategies against the disease.

## Materials and methods

### Data source and processing

#### TCGA cohort

The transcriptomic, clinical, and DNA methylation data on the Liver Hepatocellular Carcinoma (LIHC) cohort were downloaded from TCGA, which contains information on 374 tumor tissues and 50 adjacent normal tissues (Download date: August 16, 2022). DNA methylation data from 380 tumor tissues and 50 adjacent normal tissues were selected. Samples containing both transcriptomic and DNA methylation data were then screened for further analysis.

#### GEO cohort

The gene expression profiles of the GSE54236 liver cancer dataset were obtained from the GEO database (http://www.ncbi.nlm.nih.gov/geo/). GSE54236 was defined as the independent external dataset for validation of the prognostic gene signature. In total, 161 liver tissue samples (81 tumors and 80 tumor adjacent tissue samples) were included. The samples were prospectively derived from hepatocellular carcinoma tissue as well as non-tumor tissue from the livers of the same patients. Detailed clinicopathological data, including age, TNM stage, tumor grade, and survival data, were downloaded **Supplementary Table 1** lists the clinical features of the patients in the training and validation sets.

#### Data processing

For both the TCGA-LIHC and GSE54236 datasets, the expression profiles were normalized to transcripts and log2 transformed. The intersection of the expression matrices of both datasets was used to determine the expression of the intersecting genes. Simultaneously, batch correction was performed on the two datasets to obtain the corrected expression matrices. The “sva” package of R (http://www.bioconductor.org/packages) was used for this process.

### Differential expression analysis

The “limma” package of R was used to identify genes with differential expression between the tumor and adjacent normal tissues of the TCGA-LIHC dataset, where the negative binomial distribution method was applied. The thresholds were an absolute log FC value of greater than 0.585 and a false discovery rate of less than 0.05. The “pheatmap” package of R was used to plot a heatmap for visualization of the gene expression data.

### Identification of DNA methylation-driven genes

Having identified the differentially expressed genes, the ones with differential DNA methylation levels were next identified using the “MethyMix” package of R (threshold: absolute log FC > 1, *p*-value < 0.05). Then, using standard Spearman’s correlation analysis (criterion: absolute value of the correlation coefficient > 0.3), the genes whose expression levels were negatively correlated with their methylation levels were identified as DNA methylation-driven genes.

### Construction and validation of a risk score model based on DNA methylation-driven genes

Using the “survival” package of R, univariate Cox regression analysis and log-rank tests were performed to analyze the significant DNA methylation-driven genes related to prognosis. The “forestploter” package of R was used to generate a forest plot of the univariate analysis results. The TCGA data were used as the training dataset to construct a risk score model based on DNA methylation-driven genes. The least absolute shrinkage and selection operator (LASSO) of Cox regression analysis was used to determine the best weighting coefficient for those prognostic genes. After a 1000-fold cross-validation of the penalized maximum likelihood estimates, the minimum criterion was determined from the optimal value of the penalty parameter (λ), and the risk score model was finally established. Based on the significant candidate genes, the formula for calculating the risk score for each sample was as follows: risk score = (β1 × Exp1) + (β2 × Exp2) + (β3 × Exp3), where β represents the coefficient value and Exp is the gene expression level.

### Gene set enrichment analysis

To obtain a list of candidate genes for further study, gene set enrichment analysis (GSEA) of the Kyoto Encyclopedia of Genes and Genomes (KEGG) gene set “c2.cp.kegg.v7.1.symbols.gmt.xz” was carried out to extract all relevant DNA methylation-driven genes that were analyzed. The “ggplot2” package of R was used to visualize the multiple GSEA results. A KEGG pathway analysis was performed to reveal the potential underlying mechanisms of the DNA methylation-driven genes associated with the risk scores. Pathways enriched in the different DNA methylation-driven genes of the risk score datasets were evaluated using GSEA (v4.0.2 software; http://software.broadinstitute.org/gsea/login.jsp), where a *p*-value of less than 0.05 and a false discovery rate (*q*) of less than 0.25 was considered indicative of statistical significance.

### RNA extraction and qRT-PCR

We selected relevant cases according to the following inclusion and exclusion criteria. Inclusion criteria: 1. Patients who meet the diagnostic criteria for hepatocellular carcinoma in the third edition of the 2017 NCCN guidelines. 2. Aged between 18 and 70 years old, male or female;3. Enrolled subjects must have a histologically or cytologically confirmed primary or metastatic tumor that meets the criteria;4. Adequate organ and bone marrow function, without serious hematopoietic abnormality and abnormal heart, lung, liver, kidney function and immunodeficiency;5. No other malignant tumor related medical history. Exclusion criteria:1. Have severe underlying disease that is poorly controlled (e.g., malignant hypertension, congestive heart failure, unstable angina, atrial fibrillation, arrhythmia);2. Women who are lactating or pregnant;3. Those with a history of mental illness;4. Those with a history of serious infectious disease illness (e.g., infection with human immunodeficiency virus HIV, divergent bacilli of tuberculosis, etc.);5. Incapacitated persons or persons with limited ability to act.

We conducted relevant experimental verification on tumor tissue and adjacent normal tissue of 30 newly diagnosed patients who met the diagnostic criteria of hepatocellular carcinoma in the third edition of NCCN guidelines in 2017 in the First Affiliated Hospital of Sun Yat-sen University. All patients were not receiving radiotherapy or chemotherapy. At the time of sample collection, all patients were free of other serious infectious diseases (e.g., infection with human immunodeficiency virus HIV, Mycobacterium tuberculosis divergent bacilli, etc.).

Total RNA was extracted from the tumor cells using the MolPure^®^ Cell/Tissue Total RNA Kit (Cat: 19221ES50; Yeasen, Shanghai, China) and reverse-transcribed using SuperScript™ III Reverse Transcriptase (Invitrogen, Thermo Fisher Scientific, Shanghai, China) and specific primers (sequences listed in **Supplementary Table 2**). The real-time reverse transcription-polymerase chain reaction (qRT-PCR) was performed using the SYBR Green PCR Master Mix (11184ES08; Yeasen), and the PCR products were detected using standard protocols.

### Immunohistochemical staining

Target proteins were identified through semi-quantitative immunohistochemical analysis of human liver cancer tissue, using the 2-Step Plus Poly-HRP Anti-Mouse/Rabbit IgG Detection System (with DAB solution) (E-IR-R217, 6 mL; Elabscience, Wuhan, China). In brief, the tissue section was fixed with 4% cold paraformaldehyde for 15 min and then washed three times with phosphate-buffered saline. Subsequently, the tissue was blocked and then incubated with primary and secondary antibodies(Cat:37597, Human, Application concentration:1:25-1:100), following which 0.5 g/mL 4′,6-diamidino-2-phenylindole was added for nuclear staining. Then, the percentage of positive cells and staining intensity were scored under a microscope. Five high magnification fields of view (200x) were observed for each tissue section and the number of positive cells counted was scored against the intensity of positive staining, which was determined independently. If the difference is greater than 6 points, the scores are reassessed.

### Statistical analysis

Using the “survival” and “survminer” packages of R, the patients with LIHC were stratified into high- and low-risk subgroups according to their median estimated risk scores. Survival was compared between the two subgroups using Kaplan–Meier curves and log-rank tests. The prediction accuracy of the risk score model based on DNA methylation-driven genes was quantified with time-dependent receiver operating characteristic (ROC) curves, and the area under the ROC curve was calculated using the “survival ROC” package of R. Univariate and multivariate Cox regression analyses were performed to identify factors influencing overall survival, including age, sex, tumor grade, clinical stage, and risk score. All the factors identified in the univariate analyses were included in the multivariate analysis. SPSS software (v24.0; SPSS, Inc., Chicago, IL, USA) and R software (v4.1.0; R Foundation for Statistical Computing, Vienna, Austria) were used for the statistical analyses. A two-sided *p*-value of less than 0.05 was considered statistically significant.

## Results

### Identification of the differentially expressed genes

**Figure 1:**
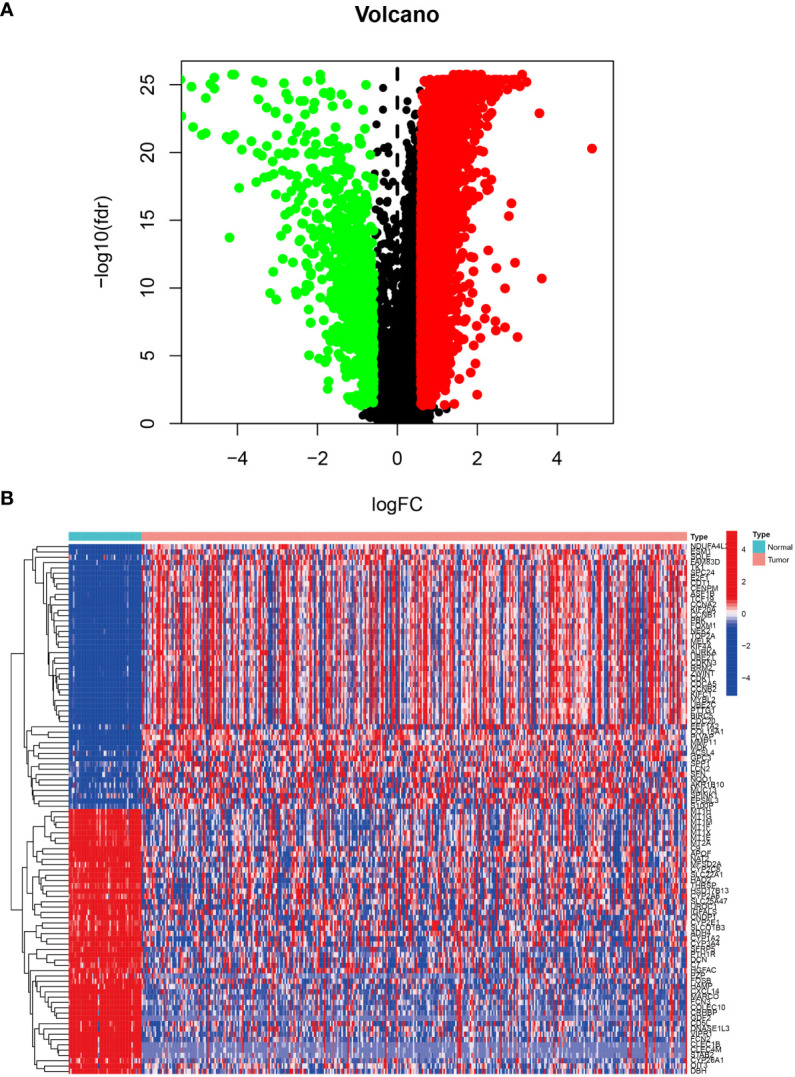
Differentially expressed genes in LIHC tissue from the TCGA database. **(A)** Volcano plot of differential gene expression in LIHC. The black dots indicate inconspicuity; red and green dots represent significant hypermethylation or hypomethylation, respectively. **(B)** Heatmap of the top 50 upregulated and downregulated genes.

The LIHC dataset downloaded from TCGA was used to examine the differences in gene expression levels between the HCC tissues and adjacent normal tissues. The volcano plot of the differentially expressed genes in LIHC is shown in **Figure 1A** and the top 50 upregulated and downregulated genes are shown in **Figure 1B**. In **Figure 1A**, the black dots indicate inconspicuity; red and green dots represent significant hypermethylation or hypomethylation, respectively. In **Figure 1B**, the upper part on the left side of the heat map shows that 50 related genes are low expressed in the adjacent normal issues, while the upper part on the right side shows that these 50 genes are highly expressed in HCC issues; The lower part on the left describes the other 50 genes that are highly expressed in adjacent normal issues, and the lower part on the right shows that these 50 genes are low expressed in HCC issues.

### DNA methylation-driven genes

**Figure 2:**
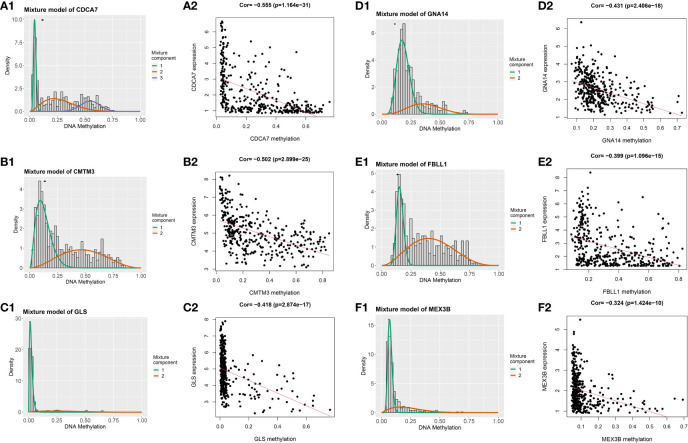
Genes with an absolute value of the correlation coefficient greater than 0.3 between their DNA methylation and gene expression levels. The differences in DNA methylation levels between the tumor and adjacent normal tissues are shown on the left **(A1, B1, C1, D1, E1, F1)**. The Mixture Component here suggests the methylation distribution of this gene in the tumor, with 1 and 2 representing two different distributions, with the peaks both to the left of the black line normal sample, indicating that this gene is hypermethylated in the normal sample and hypomethylated in the tumor sample. The histogram demonstrates the distribution of methylation in tumor samples. Horizontal black bars show the distribution of methylation in normal samples. The results of the correlation analysis between gene expression and DNA methylation levels are on the right **(A2, B2, C2, D2, E2, F2)**.

**Figure 3:**
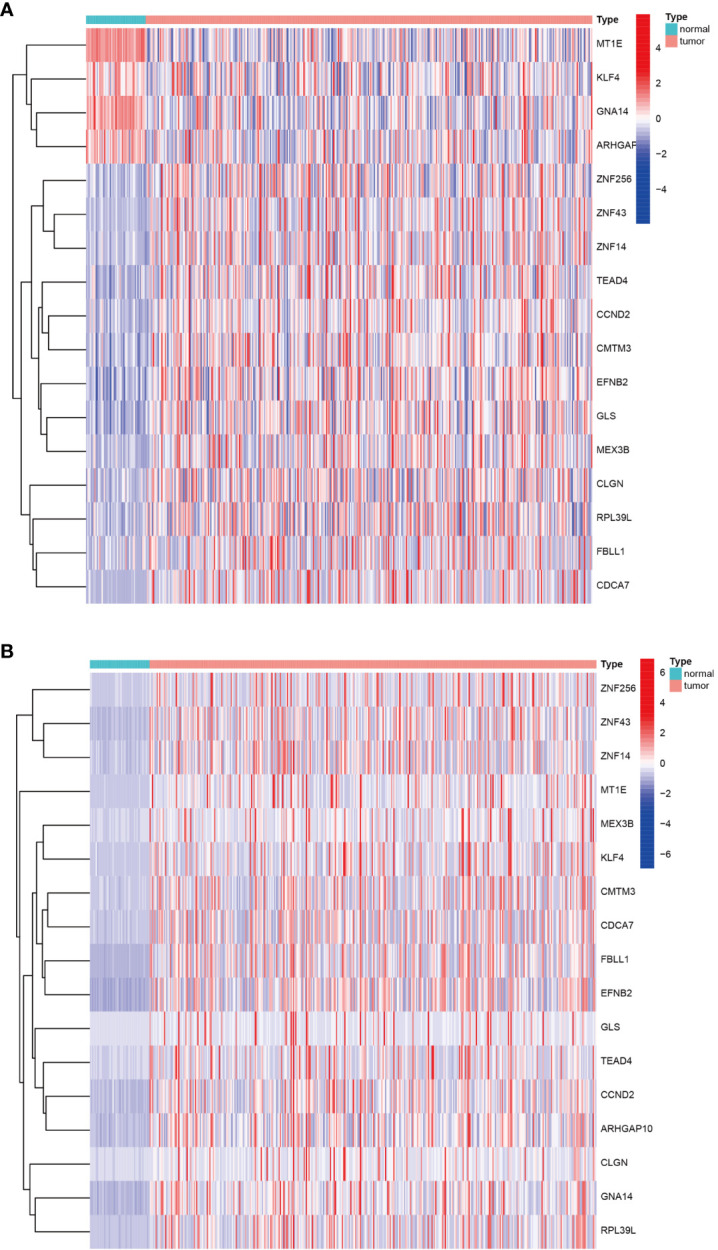
Heatmap of DNA methylation-driven genes. **(A)** Heatmap of the DNA methylation levels of 17 DNA methylation-driven genes. **(B)** Heatmap of the mRNA expression levels of 17 DNA methylation-driven genes.

Having identified the differentially expressed genes, the ones with differential DNA methylation levels were next identified using the “MethyMix” package of R. In total, 17 DNA methylation-driven genes were identified, encoding cell division cycle associated 7 (CDCA7), CKLF like MARVEL transmembrane domain containing 3 (CMTM3), glutaminase (GLS), G protein subunit alpha 14 (GNA14A), fibrillarin like 1 (FBLL1), mex-3 RNA-binding family member B (MEX3B), Rho GTPase-activating protein 10 (ARHGAP10), et al. The absolute value of the correlation coefficient between the gene expression and methylation levels was greater than 0.3 for all identified genes (**Figure 2**). From **Figure 2**, we can clearly see that the left side (A1-F1) shows the difference in DNA methylation levels between the tumor and the adjacent normal tissue, suggesting that this gene is hypermethylated in the normal sample and hypomethylated in the tumor sample. The results of the correlation analysis between gene expression and DNA methylation levels are on the right(A2-F2). The right is a statistically significant negative correlation between the level of methylation modification and the expression level of these genes. Several of the 17 DNA methylation-driven genes are shown in **Supplementary Figure 1**. Heatmaps of the DNA methylation and mRNA levels of these 17 genes are shown in **Figure 3**. We can clearly see in **Figure 3** reflecting the DNA methylation levels, the first four genes (MT1E,KLF4,GNA14,ARHGAP) are highly expressed in normal tissues and lowly expressed in tumor tissues. **Figure 3B** reflects the mRNA expression levels, and all 17 genes were highly expressed in tumor tissues and lowly expressed in normal tissues.

### Construction of the prognostic risk score model

**Figure 4:**
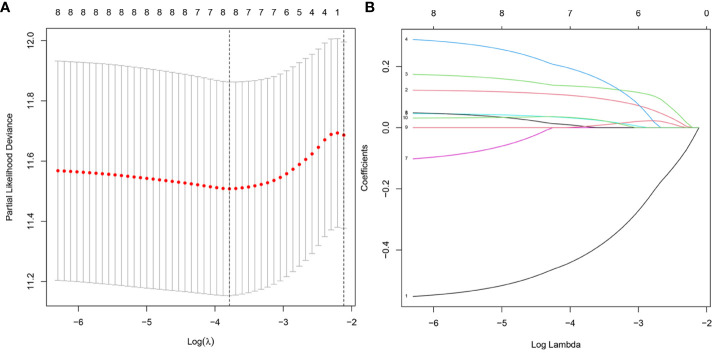
Model development for DNA methylation-driven genes. **(A)** One thousand bootstrap repeats using LASSO Cox regression for variable selection. **(B)** LASSO coefficients for genes that are DNA methylation driven. Each curve shows a gene driven by methylation(MT1E, GNA14, FBLL1, GLS, EFNB2, CCND2, MEX3B, CLGN).The end of the gene will point to a vertical coordinate which is the coefficient value.

We validate the model accuracy by using TCGA database as Training cohort, GEO database as Validation cohort. The model construction was used to obtain the model formula. In our constructed model, the patient’s risk score is equal to the expression of this gene multiplied by its corresponding coefficient. Of the 17 DNA methylation-driven genes, 10 were identified as being associated with prognosis (**Supplementary Figure 2**). LASSO Cox regression analysis was performed with 1000 bootstrap replicates to obtain the penalized maximum likelihood estimates (**Figure 4A**). The optimal weighting coefficient of each gene was determined from the regularization parameter (λ) using the 1–SE standard (**Figure 4B**). Four genes with high coefficients were selected for the development of the risk score model. The formula for calculating the risk score was as follows: risk score = (0.1449 × expression of *FBLL1*) – (0.5451 × expression of *GNA14*) + (0.1927 × expression of *GLS*) + (0.2789 × expression of *MEX3B*). The risk score was calculated for the TCGA and GSE54236 datasets, respectively, and the patients were then stratified into high- and low-risk groups according to the median score of the corresponding dataset.

### Validation of the prognostic efficiency of the risk score model

**Figure 5:**
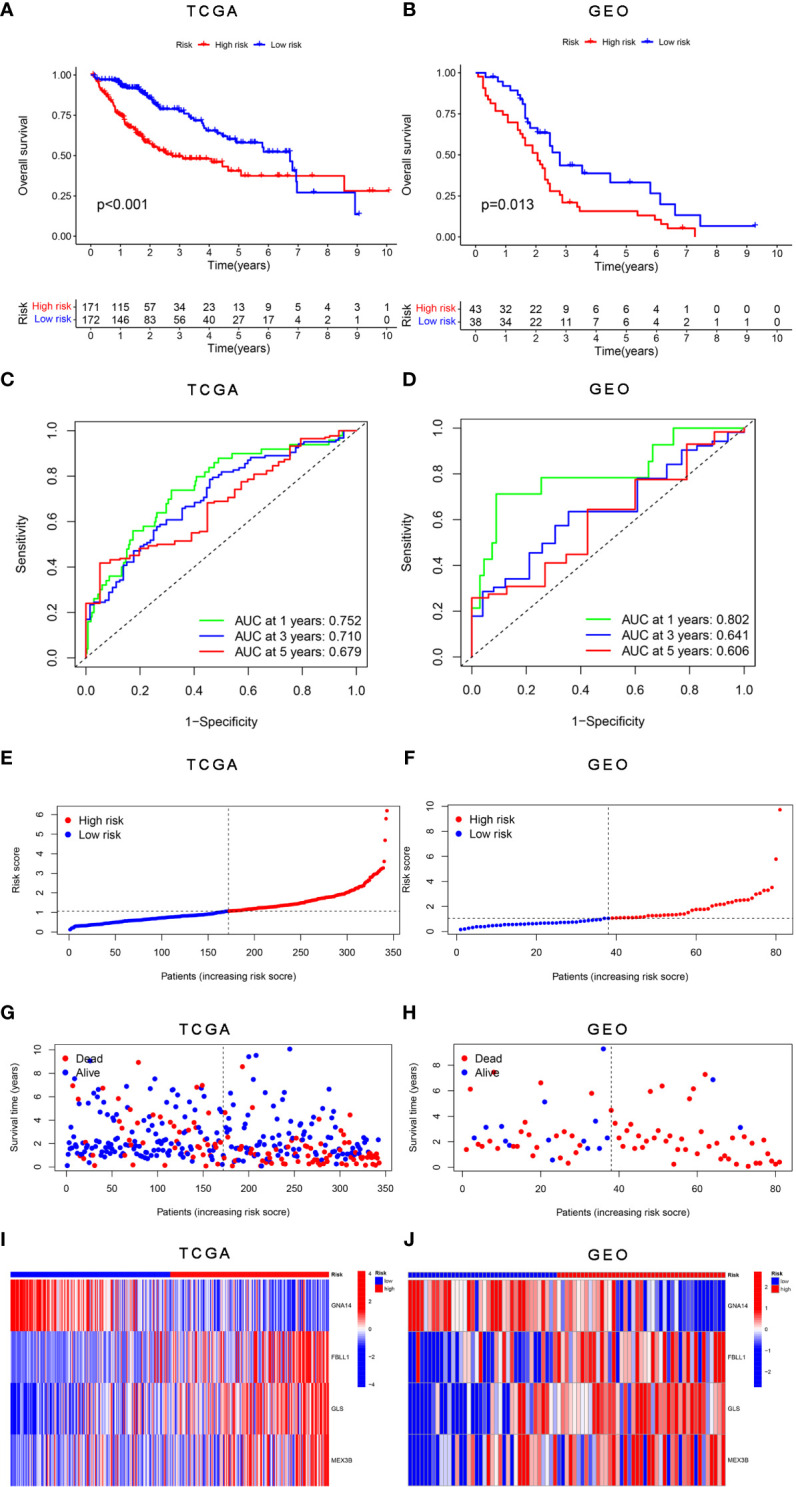
Validation of the risk score model for DNA methylation-driven genes. **(A, B)** Differences in survival between the high-risk and low-risk groups in each dataset. **(C, D)** Through time-dependent ROC analysis, the sensitivity and specificity of the risk score model based on DNA methylation-driven genes were evaluated for each dataset. **(E–H)** Dot plots comparing the outcomes of the patients in the high-risk and low-risk groups. **(I, J)** Heatmap depicting the outcomes for the three genes in both the training and validation cohorts. **(A, C, E, G)** represent TCGA, whereas **(B, D, F, H, J)** represent GSE54236.

The survival analysis results showed that patients in the high-risk group had worse survival than those in the low-risk group in both the training (**Figure 5A**) and validation cohorts (**Figure 5B**). The sensitivity and specificity of the risk score model were assessed through time-dependent ROC curve analysis. The areas under the ROC curves for 1-, 3-, and 5-year survival were 0.752, 0.710, and 0.679, respectively, for the TCGA training dataset (**Figure 5C**) and 0.802, 0.641, and 0.606, respectively, for the GSE54236 validation dataset (**Figure 5D**). **Figures 5E, F** shows the results of the analysis of the data from the TCGA and GSE54236 database for the patient increasing risk scores in the high- and low-risk group. We also created dot plots to compare the survival of patients in the high- and low-risk groups and found that the latter group had a better survival rate in both datasets (**Figures 5G, H**). We used heatmaps to compare the expression of the four DNA methylation-driven genes between the TCGA and GSE54236 datasets (**Figures 5I, J**), whereupon it was found that their expression levels were slightly different in both datasets but relatively consistent.

### Univariate and multivariate Cox regression analyses

**Figure 6:**
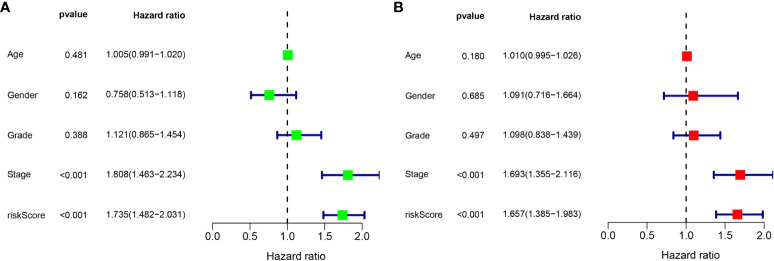
Univariate and multivariate Cox regression analyses. **(A)** Results of the univariate analysis. **(B)** Results of the multivariate analysis.

**Figure 7:**
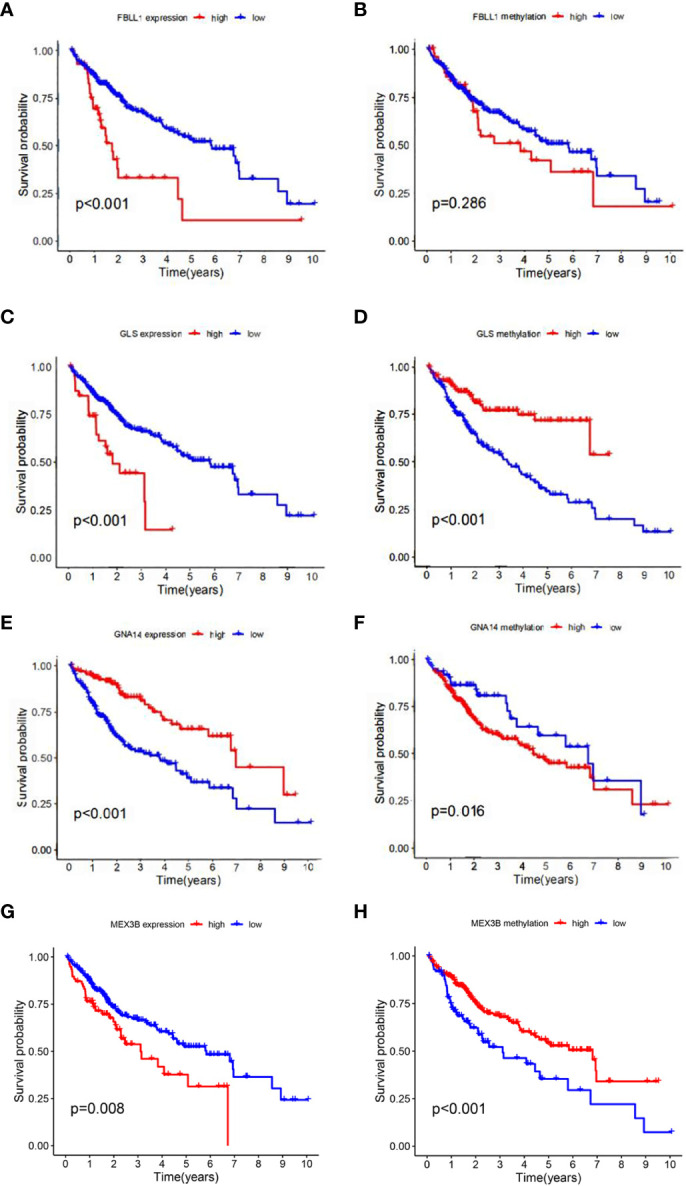
Four genes involved in model construction and the DNA methylation and gene expression levels predict survival in the general population. **(A, C, E, G)** Kaplan–Meier survival analysis of the respective effects of *FBLL1*, *GLS*, *GNA14*, and *MEX3B* expression on overall survival. **(B, D, F, H)** Kaplan–Meier survival analysis of the respective effects of the DNA methylation levels of *FBLL1*, *GLS*, *GNA14*, and *MEX3B* on overall survival.

According to the results of the univariate Cox regression analysis, the clinical stage and risk score were significantly associated with overall survival (**Figure 6A**). In the multivariate Cox regression analysis, the clinical stage and risk score were also independently associated with overall survival (**Figure 6B**). Additionally, the effects of *FBLL1*, *GLS*, *GNA14*, and *MEX3B* expression on survival were investigated through Kaplan–Meier survival analysis of the TCGA cohort. It was found that low *GNA14* expression (**Figure 7E**) and high *FBLL1*, *GLS*, and *MEX3B* expression (**Figures 7A, C, G**) were associated with poor overall survival. Subsequently, we analyzed the association of the DNA methylation levels of these four important genes with overall survival, whereupon it was found that hypomethylation of *GLS* and *MEX3B* (**Figures 7D, H**) and hypermethylation of *GNA14* (**Figure 7F**) were associated with poor overall survival. However, because there was no difference between the DNA methylation level of *FBLL1* and overall survival (*p* > 0.05), this gene could be excluded from further analysis (**Figure 7B**).

### Gene set enrichment analyses

**Figure 8:**
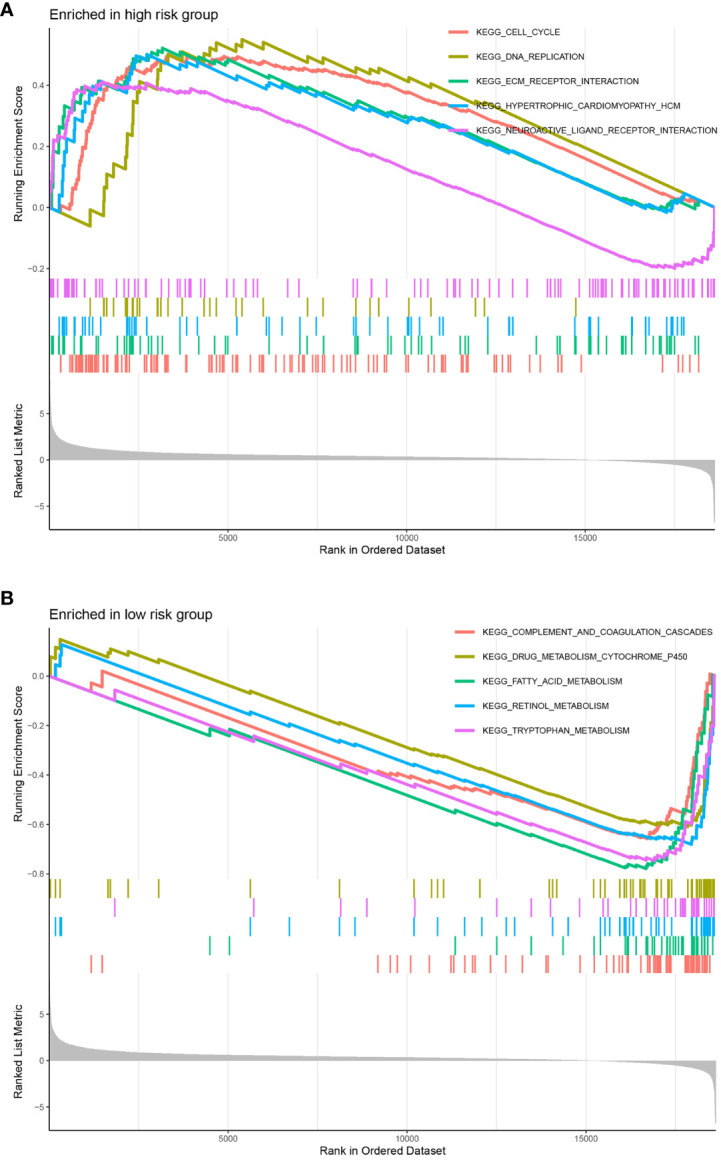
Significantly enriched pathways in each dataset. The top 5 pathways were enriched in the high-risk group **(A)** compared with the low-risk group **(B)**. The vertical coordinate represents the scores, and the different color curves represent different pathways.

We performed GSEA on each dataset to explore the KEGG pathways enriched in the DNA methylation-driven genes as well as other pathways associated with covariates of those genes. The pathways with significant gene enrichment were concentrated in the high-risk group and were mostly related to DNA methylation. These included the cell cycle, DNA replication, extracellular matrix–receptor interaction, hypertrophic cardiomyopathy, and neuroactive ligand-receptor interaction pathways (**Figure 8A**). The pathways with significant gene enrichment that were concentrated in the low-risk groups are shown in **Figure 8B**.

### qRT-PCR verification of gene expression and immunohistochemistry staining

**Figure 9:**
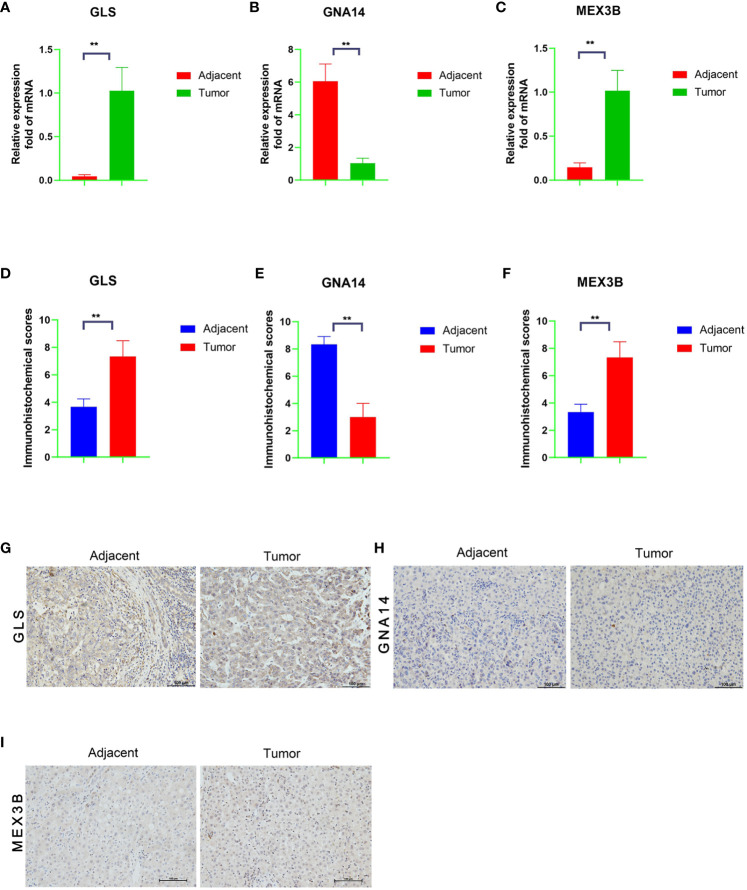
mRNA and protein expression levels of the three DNA methylation-driven genes. **(A–C)** mRNA levels of the target genes in the tumor and adjacent normal tissues, as determined by qRT-PCR assay. **(G–I)** Protein levels in the tumor and adjacent normal tissues, as determined by immunohistochemistry, and a Score Statistics Chart **(D–F)**.

We performed qRT-PCR verification of the expression of the three genes in tumor tissues and adjacent normal tissues of 30 patients with liver cancer. The *GNA14* expression level was found to be higher in the adjacent tissues than in the tumor tissues, whereas *GLS* and *MEX3B* expression was lower in the adjacent normal tissues (**Figures 9A–C**). Subsequently, we conducted further immunohistochemical staining of the two types of tissue and obtained results consistent with the qRT-PCR findings (**Figures 9D–I**). These results indicate that *GNA14* is a tumor suppressor gene, whereas *GLS* and *MEX3B* can be considered tumorigenic oncogenes.

## Discussion

At present, the mortality rate of patients with liver cancer remains high worldwide. Many afflicted patients are diagnosed only at the advanced disease stages, which makes the development of reliable prognostic and survival prediction methods especially important. Using both the TCGA-LIHC and GSE54236 datasets, we created a new prognostic risk score model based on DNA methylation-driven genes. Independent datasets were used to validate the sensitivity and specificity of the model. In the multivariate analyses, the DNA methylation-based risk score was found to be an independent prognostic factor. Lastly, a mixed nomogram model with a DNA methylation-based risk score was created to predict overall survival (**Supplementary Figure 3**).We found that disease stage and risk score are the main factors affecting the prognosis.

DNA methylation plays an important role in tumorigenesis (22, 23). The expression of tumor suppressor genes or oncogenes can be controlled by increasing or decreasing the DNA methylation of the target gene, which affects the incidence and growth of tumors (24). In this study, the mRNA levels of *GNA14* were found to be negatively correlated with its DNA methylation levels, and the hypermethylation of this gene was associated with poor overall survival. *GNA14*, a tumor suppressor gene, is hypermethylated in hepatitis B virus-related HCC (25). In that study, DNA methylation was determined to be the cause of altered *GNA14* expression and was regulated by the hepatitis B virus X (HBx) protein. Mechanistically, GNA14 likely controls the retinoblastoma pathway by inducing Notch1 cleavage to stop tumor growth, and it may also prevent tumor cell migration by suppressing the expression of Jumonji domain-containing 6 (JMJD6) (25).

At both the genetic and epigenetic levels, the importance of DNA methylation in cancer occurrence and development is beyond doubt. During the development of liver cancer, liver fibrosis is a common pathological change that accompanies the progress of liver cancer. Persistent liver fibrosis will eventually lead to the occurrence of liver cancer. The transformation of hepatic stellate cells into hepatic fibroblasts is an important process in the process of liver fibrosis. During this period, gene expression in cells is regulated by DNA methylation. When abnormal methylation is inhibited, the fibroblast-like transformation of hepatic stellate cells can be reversed (26). Angiogenesis in liver cancer can promote the proliferation and metastasis of cancer cells, while abnormal DNA methylation significantly affects angiogenesis in liver cancer. Research shows that chymotrypsin 1, tyrosine kinase non-receptor 2 and transforming growth factor are up-regulated in liver cancer cells through gene hypomethylation β Receptor II promotes tumor angiogenesis (27, 28). In addition, HBV infection can induce the occurrence of DNA abnormal methylation in liver cancer. Relevant studies show that P16, RASSF1A, GSTP1, APC, p15 and SFRP1 genes are significantly hypermethylation in HBV positive liver cancer (29).

We found that high *GLS*, *FBLL1*, and *MEX3B* expressions were associated with poor overall survival, as was the hypomethylation of *GLS* and *MEX3B.* The theoretical basis behind the development of metabolic therapies is that altered glutamine metabolism is a hallmark of cancer progression. GLS is a critical enzyme that controls glutamine metabolism (30). Furthermore, it was found that GLS knockdown severely reduced Wnt/-catenin pathway activity, and its knockout caused apoptosis and cell cycle arrest (31).

*FBLL1* encodes a protein that is related to RNA binding and methyltransferase activities, according to Gene Ontology annotation. An important paralog of this gene is *FBL*, which is closely associated with tumors (32).

With regard to *MEX3B*, our results are in line with those of previous research on this gene. According to the TCGA melanoma dataset, higher MEX3B expression is linked to lower cytolytic activity and reduced lymphocyte infiltration in patients with this type of skin cancer. Increased MEX3B expression has also been associated with melanoma resistance to anti-PD-1 immunotherapy (33–36). These results indicate that MEX3B is closely related to tumorigenesis and cancer development.

Glutaminase (GLS), a crucial enzyme involved in the regulation of glutamine metabolism, has been reported to play crucial roles in cancer development (37–39). High expression of GLS was significantly associated with Gleason score and Tumor stage. Glutamine, which is the most important source of nitrogen and respiratory fuel for tumor cells (40). It plays a key role in tumor metabolism process, and it can inhibit tumor cells by preventing and disturbing the metabolism of glutamine in tumor cells. Although glutamine is a non-essential amino acid, it is essential for rapidly proliferating tumor cells and can provide nitrogen, carbon and energy for the synthesis of macromolecular materials. Hepatoma cells contain a large number of glutaminase, which is the initiating enzyme and the key enzyme (rate-limiting enzyme) of tumor cells using glutamine for glutamine yeast (41). Hypermethylation-mediated downregulation of CPS 1 (carbamoyl phosphosynthetase I), a liver-specific rate-limiting enzyme of the urea cycle, was reported as an HCC-hypermethylated gene (42). It may facilitate the use of glutamine by CAD (carbamoyl phosphate synthetase II) in HCC, which initiates a pathway of pyrimidine synthesis for cell division. The mean level of CAD RNA in HCC was reported to increase 2.8-fold compared with normal liver tissue (p = 6.7×10−34), while the mean level of CPS1 RNA was reduced 2.1-fold compared with normal liver tissue (43). Therefore, glutaminase has an important role in tumor-associated metabolic reprogramming (increased glutaminolysis) and may be related to the growth and malignancy of tumor cells.

Nomograms are user-friendly graphical composite models that are more accurate than traditional staging techniques in predicting prognosis in various types of cancer (44). A nomogram may calculate the likelihood of an event occurring based on specific patient facts, such as survival and recurrence. Using *GLS*, *FBLL1*, *MEX3B*, and *GNA14*, a nomogram with excellent discriminative ability was created to assess the survival risk of each patient. This renders it possible for our gene signature to be used for making more accurate and pertinent predictions in the clinical setting.

The evaluation of significantly mutated genes in human tumors is essential for cancer diagnosis and treatment and reasonable therapy selection (45, 46). Previous studies have demonstrated that TP53 mutations occur in several types of cancer and decrease antitumor immune responses (47, 48). DNA methylation selectively methylates cytosine, one of the building blocks of DNA (49). This modification changes the shape of the DNA chain, causing some proteins to fail to recognize it properly, and can lead to some gene functions being abnormally “turned on” or “turned off.” In studies on mice infected with viruses or bearing tumors, the T cells were found to be depleted because the genes in the cells were turned off by DNA methylation, and the depleted state was passed on to the next generation of T cells. It was also found that decitabine, a commonly used cancer chemotherapy drug, reversed this complete depletion of T cells. In tumor-bearing mice treated with the drug, followed by immunotherapy, the T cells “reactivated in place,” regained their fighting power, and proliferated (50). These findings could help toward the development of immunotherapies with improved efficacy against cancer and chronic viral infections.

Despite the inclusion of several regulatory factors and experimental verification of the prognostic risk score model, our study had some limitations. We did not conduct further studies using animal models or related clinical trials to explore the specific regulatory mechanisms of these genes.

DNA methylation is one of the most important epigenetic modifications and plays a crucial role in carcinogenesis. In this study, we identified three DNA methylation-driven gene markers unique to HCC; namely, *GNA14*, *GLS* and *MEX3B*. Immunohistochemical and qRT-PCR assays revealed that the *GLS* and *MEX3B* expression levels were significantly decreased in human HCC tissue. The developed risk score model indicates that these gene markers have a high degree of sensitivity and specificity in differentiating cancerous tissue from normal tissue.

## Conclusions

In conclusion, we have developed a gene signature based on genes associated with DNA methylation and a predictive nomogram of overall survival for patients with HCC, both of which will be highly useful in therapeutic settings. Additionally, we believe that our study findings will help toward elucidating the cellular and biological mechanisms underlying the occurrence and progression of liver cancer, improving the early detection and intervention of cancer in clinical practice, and aiding the development of new potential therapeutic targets as well as methods for the prevention of cancer metastasis.

## Data availability statement

The original contributions presented in the study are included in the article/**Supplementary Material**. Further inquiries can be directed to the corresponding authors.

## Ethics statement

The studies involving humans were approved by Committee on the Use of Clinical Research and Animal Trials of the First Affiliated Hospital of Sun Yat-sen University. The studies were conducted in accordance with the local legislation and institutional requirements. The participants provided their written informed consent to participate in this study.

## Author contributions

JL designed the experiments, analyzed the data, and prepared the manuscript. S-JZ, B-JH, and C-FY conducted the experiments. W-CZ and Q-XC devised the methodology, collected data, and provided the specimens. All authors contributed to the article and approved the submitted version.

## Supplementary material

Supplementary Table 1 Clinicopathologic characteristics of patients in TCGA and GEO cohorts.

Supplementary Table 2 Primers for the qRT-PCR assay.

Supplementary Figure 1 Several of the 17 DNA methylation-driven genes.

Supplementary Figure 2 10 DNA methylation-driven geneswere identified as being associated with prognosis.

Supplementary Figure 3 Building the combined nomogram to predict the overall survival (OS) of patients with HCC.

## Glossary

**Table:**

HCC	Hepatocellular carcinoma
TERT	Telomerase reverse transcriptase
TP53	Tumor protein p53
CTNNB1	Catenin beta 1
BRCA1	Breast cancer gene 1
TMS1	Target of methylation-induced silencing 1
DAPK	Death-associated protein kinase
TCGA	The Cancer Genome Atlas
GEO	Gene Expression Omnibus
FC	Fold-change
LIHC	Liver Hepatocellular Carcinoma
GSEA	Gene set enrichment analysis
KEGG	Kyoto Encyclopedia of Genes and Genomes
qRT-PCR	Quantitative Real Time polymerase chain reaction
ROC	Receiver operating characteristic
CDCA7	Cell division cycle associated 7
CMTM3	CKLF like MARVEL transmembrane domain containing 3
GLS	Glutaminase
GNA14A	G protein subunit alpha 14
FBLL1	Fibrillarin like 1
MEX3B	Mex-3 RNA-binding family member B
ARHGAP10	Rho GTPase-activating protein 10
CCND2	Cyclin D2
CLGN	Calmegin
EFNB2	Ephrin B2
KLF4	KLF transcription factor 4
TEAD4	TEA domain transcription factor 4
MT1E	Metallothionein 1E
RPL39L	Ribosomal protein L39 like
ZNF	Zinc finger protein
HBx	Hepatitis B virus X
JMJD6	Jumonji domain-containing 6
CPS 1	Carbamoyl phosphosynthetase I

## References

[1] Khare S Khare T Ramanathan R Ibdah JA. Hepatocellular carcinoma: the role of MicroRNAs. Biomolecules (2022) 12:4–5. doi: 10.3390/biom12050645 PMC9138333 35625573

[2] Marrero JA. Hepatocellular carcinoma. Curr Opin Gastroenterol (2006) 22:248–53. doi: 10.1097/01.mog.0000218961.86182.8c 16550039

[3] Hanahan D. Hallmarks of cancer: new dimensions. Cancer Discovery (2022) 12:31–46. doi: 10.1158/2159-8290.CD-21-1059 35022204

[4] Chidambaranathan-Reghupaty S Fisher PB Sarkar D. Hepatocellular carcinoma (HCC): Epidemiology, etiology and molecular classification. Adv Cancer Res (2021) 149:1–61. doi: 10.1016/bs.acr.2020.10.001 33579421 PMC8796122

[5] Chakraborty E Sarkar D. Emerging therapies for hepatocellular carcinoma (HCC). Cancers (Basel) (2022) 14:10–11. doi: 10.3390/cancers14112798 PMC9179883 35681776

[6] Li S Tollefsbol TO. DNA methylation methods: Global DNA methylation and methylomic analyses. Methods (2021) 187:28–43. doi: 10.1016/j.ymeth.2020.10.002 33039572 PMC7914139

[7] Kulis M Esteller M. DNA methylation and cancer. Adv Genet (2010) 70:27–56. doi: 10.1016/B978-0-12-380866-0.60002-2 20920744

[8] Bock C. Analysing and interpreting DNA methylation data. Nat Rev Genet (2012) 13:705–19. doi: 10.1038/nrg3273 22986265

[9] Liu XS Wu H Ji X Stelzer Y Wu X Czauderna S. Editing DNA methylation in the mammalian genome. Cell (2016) 167:233–47. doi: 10.1016/j.cell.2016.08.056 PMC5062609 27662091

[10] Thakur C Chen B Li L Zhang Q Yang ZQ Chen F. Loss of mdig expression enhances DNA and histone methylation and metastasis of aggressive breast cancer. Signal Transduct Target Ther (2018) 3:25. doi: 10.1038/s41392-018-0027-4 30254753 PMC6147911

[11] Kulis M Merkel A Heath S Queiros AC Schuyler RP Castellano G. Whole-genome fingerprint of the DNA methylome during human B cell differentiation. Nat Genet (2015) 47:746–56. doi: 10.1038/ng.3291 PMC5444519 26053498

[12] Smith ZD Meissner A. DNA methylation: roles in mammalian development. Nat Rev Genet (2013) 14:204–20. doi: 10.1038/nrg3354 23400093

[13] Ai T Zhang J Wang X Zheng X Qin X Zhang Q. DNA methylation profile is associated with the osteogenic potential of three distinct human odontogenic stem cells. Signal Transduct Target Ther (2018) 3:1. doi: 10.1038/s41392-017-0001-6 29527327 PMC5837092

[14] Shen C Wang K Deng X Chen J. DNA N(6)-methyldeoxyadenosine in mammals and human disease. Trends Genet (2022) 38:454–67. doi: 10.1016/j.tig.2021.12.003 PMC9007851 34991904

[15] Robertson KD. DNA methylation and human disease. Nat Rev Genet (2005) 6:597–610. doi: 10.1038/nrg1655 16136652

[16] Hao X Luo H Krawczyk M Wei W Wang W Wang J. DNA methylation markers for diagnosis and prognosis of common cancers. Proc Natl Acad Sci U.S.A. (2017) 114:7414–19. doi: 10.1073/pnas.1703577114 PMC5514741 28652331

[17] Liu YN Liu Y Lee HJ Hsu YH Chen JH. Activated androgen receptor downregulates E-cadherin gene expression and promotes tumor metastasis. Mol Cell Biol (2008) 28:7096–108. doi: 10.1128/MCB.00449-08 PMC2593382 18794357

[18] Licchesi JD Westra WH Hooker CM Herman JG. Promoter hypermethylation of hallmark cancer genes in atypical adenomatous hyperplasia of the lung. Clin Cancer Res (2008) 14:2570–78. doi: 10.1158/1078-0432.CCR-07-2033 18451218

[19] Lee S Hwang KS Lee HJ Kim JS Kang GH. Aberrant CpG island hypermethylation of multiple genes in colorectal neoplasia. Lab Invest (2004) 84:884–93. doi: 10.1038/labinvest.3700108 15122305

[20] Qureshi SA Bashir MU Yaqinuddin A. Utility of DNA methylation markers for diagnosing cancer. Int J Surg (2010) 8:194–98. doi: 10.1016/j.ijsu.2010.02.001 20139036

[21] Farkas SA Milutin-Gasperov N Grce M Nilsson TK. Genome-wide DNA methylation assay reveals novel candidate biomarker genes in cervical cancer. Epigenetics (2013) 8:1213–25. doi: 10.4161/epi.26346 24030264

[22] Koch A Joosten SC Feng Z de Ruijter TC Draht MX Melotte V. Author Correction: Analysis of DNA methylation in cancer: location revisited. Nat Rev Clin Oncol (2018) 15:467. doi: 10.1038/s41571-018-0028-9 29713045

[23] Klutstein M Nejman D Greenfield R Cedar H. DNA methylation in cancer and aging. Cancer Res (2016) 76:3446–50. doi: 10.1158/0008-5472.CAN-15-3278 27256564

[24] Morgan AE Davies TJ Mc AM. The role of DNA methylation in ageing and cancer. Proc Nutr Soc (2018) 77:412–22. doi: 10.1017/S0029665118000150 29708096

[25] Song G Zhu X Xuan Z Zhao L Dong H Chen J. Hypermethylation of GNA14 and its tumor-suppressive role in hepatitis B virus-related hepatocellular carcinoma. Theranostics (2021) 11:2318–33. doi: 10.7150/thno.48739 PMC7797690 33500727

[26] Mann J Oakley F Akiboye F Elsharkawy A Thorne AW Mann DA. Regulation of myofibroblast transdifferentiation by DNA methylation and MeCP2: implications for wound healing and fibrogenesis. Cell Death Differ (2007) 14:275–85. doi: 10.1038/sj.cdd.4401979 16763620

[27] Phillips JM Goodman JI. Identification of genes that may play critical roles in phenobarbital (PB)-induced liver tumorigenesis due to altered DNA methylation. Toxicol Sci (2008) 104:86–99. doi: 10.1093/toxsci/kfn063 18359763

[28] Phillips JM Goodman JI. Multiple genes exhibit phenobarbital-induced constitutive active/androstane receptor-mediated DNA methylation changes during liver tumorigenesis and in liver tumors. Toxicol Sci (2009) 108:273–89. doi: 10.1093/toxsci/kfp031 PMC2664694 19233941

[29] Zhang C Huang C Sui X Zhong X Yang W Hu X. Association between gene methylation and HBV infection in hepatocellular carcinoma: A meta-analysis. J Cancer (2019) 10:6457–65. doi: 10.7150/jca.33005 PMC6856736 31772678

[30] Mukha A Kahya U Linge A Chen O Lock S Lukiyanchuk V. GLS-driven glutamine catabolism contributes to prostate cancer radiosensitivity by regulating the redox state, stemness and ATG5-mediated autophagy. Theranostics (2021) 11:7844–68. doi: 10.7150/thno.58655 PMC8315064 34335968

[31] Zhang J Mao S Guo Y Wu Y Yao X Huang Y. Inhibition of GLS suppresses proliferation and promotes apoptosis in prostate cancer. Biosci Rep (2019) 39:5–6. doi: 10.1042/BSR20181826 PMC6591571 31196962

[32] Wang L Zhou N Qu J Jiang M Zhang X. Identification of an RNA binding protein-related gene signature in hepatocellular carcinoma patients. Mol Med (2020) 26:125. doi: 10.1186/s10020-020-00252-5 33297932 PMC7727152

[33] Yang Y Wang SY Huang ZF Zou HM Yan BR Luo WW. The RNA-binding protein Mex3B is a coreceptor of Toll-like receptor 3 in innate antiviral response. Cell Res (2016) 26:288–303. doi: 10.1038/cr.2016.16 26823206 PMC4783467

[34] Huang L Malu S McKenzie JA Andrews MC Talukder AH Tieu T. The RNA-binding protein MEX3B mediates resistance to cancer immunotherapy by downregulating HLA-A expression. Clin Cancer Res (2018) 24:3366–76. doi: 10.1158/1078-0432.CCR-17-2483 PMC9872773 29496759

[35] Takada H Kawana T Ito Y Kikuno RF Mamada H Araki T. The RNA-binding protein Mex3b has a fine-tuning system for mRNA regulation in early Xenopus development. Development (2009) 136:2413–22. doi: 10.1242/dev.029165 19542354

[36] Le Borgne M Chartier N Buchet-Poyau K Destaing O Faurobert E Thibert C. The RNA-binding protein Mex3b regulates the spatial organization of the Rap1 pathway. Development (2014) 141:2096–107. doi: 10.1242/dev.108514 24803656

[37] Luan W Zhou Z Zhu Y Xia Y Wang J Xu B. miR-137 inhibits glutamine catabolism and growth of Malignant melanoma by targeting glutaminase. Biochem Biophys Res Commun (2018) 495:46–52. doi: 10.1016/j.bbrc.2017.10.152 29097210

[38] Li HJ Li X Pang H Pan JJ Xie XJ Chen W. Long non-coding RNA UCA1 promotes glutamine metabolism by targeting miR-16 in human bladder cancer. Jpn J Clin Oncol (2015) 45:1055–63. doi: 10.1093/jjco/hyv132 26373319

[39] Momcilovic M Bailey ST Lee JT Fishbein MC Magyar C Braas D. Targeted inhibition of EGFR and glutaminase induces metabolic crisis in EGFR mutant lung cancer. Cell Rep (2017) 18:601–10. doi: 10.1016/j.celrep.2016.12.061 PMC5260616 28099841

[40] Souba WW. Cytokine control of nutrition and metabolism in critical illness. Curr Probl Surg (1994) 31:577–643. doi: 10.1016/0011-3840(94)90047-7 7517814

[41] Lohmann R Souba WW Bode BP. Rat liver endothelial cell glutamine transporter and glutaminase expression contrast with parenchymal cells. Am J Physiol (1999) 276:G743–50. doi: 10.1152/ajpgi.1999.276.3.G743 10070052

[42] Liu H Dong H Robertson K Liu C. DNA methylation suppresses expression of the urea cycle enzyme carbamoyl phosphate synthetase 1 (CPS1) in human hepatocellular carcinoma. Am J Pathol (2011) 178:652–61. doi: 10.1016/j.ajpath.2010.10.023 PMC3069978 21281797

[43] Wheeler DA Roberts LR. Comprehensive and integrative genomic characterization of hepatocellular carcinoma. Cell (2017) 169:1327–41. doi: 10.1016/j.cell.2017.05.046 PMC5680778 28622513

[44] Sternberg CN. Are nomograms better than currently available stage groupings for bladder cancer? J Clin Oncol (2006) 24:3819–20. doi: 10.1200/JCO.2006.07.1290 16864852

[45] Wu XX Xue LG Zhao LD Wang Y Cai ZM. Effect of interferon combined with thalidomid on HEL cell apoptosis and JAK2V617F mutation gene expression. Zhongguo Shi Yan Xue Ye Xue Za Zhi (2016) 24:998–1002. doi: 10.7534/j.issn.1009-2137.2016.04.007 27531763

[46] Ow GS Ivshina AV Fuentes G Kuznetsov VA. Identification of two poorly prognosed ovarian carcinoma subtypes associated with CHEK2 germ-line mutation and non-CHEK2 somatic mutation gene signatures. Cell Cycle (2014) 13:2262–80. doi: 10.4161/cc.29271 PMC4111681 24879340

[47] Wu X Lv D Cai C Zhao Z Wang M Chen W. A TP53-associated immune prognostic signature for the prediction of overall survival and therapeutic responses in muscle-invasive bladder cancer. Front Immunol (2020) 11:590618. doi: 10.3389/fimmu.2020.590618 33391264 PMC7774015

[48] Long J Wang A Bai Y Lin J Yang X Wang D. Development and validation of a TP53-associated immune prognostic model for hepatocellular carcinoma. Ebiomedicine (2019) 42:363–74. doi: 10.1016/j.ebiom.2019.03.022 PMC6491941 30885723

[49] Chiappinelli KB Strissel PL Desrichard A Li H Henke C Akman B. Inhibiting DNA Methylation Causes an Interferon Response in Cancer *via* dsRNA Including Endogenous Retroviruses. Cell (2017) 169:361. doi: 10.1016/j.cell.2017.03.036 28388418

[50] You L Han Q Zhu L Zhu Y Bao C Yang C. Decitabine-mediated epigenetic reprograming enhances anti-leukemia efficacy of CD123-targeted chimeric antigen receptor T-cells. Front Immunol (2020) 11:1787. doi: 10.3389/fimmu.2020.01787 32973749 PMC7461863

