# Supplementary material for: A prognostic model based on DNA methylation-related gene expression for predicting overall survival in hepatocellular carcinoma
Source: Front Oncol. 2024 Jan 18;13:1171932. doi: 10.3389/fonc.2023.1171932 (PMC10830715; doi:10.3389/fonc.2023.1171932)
Supplement: Supplementary Table 1 — Clinicopathologic characteristics of patients in TCGA and GEO cohorts. [file Table_1.docx]

**Table S1**

| Characteristics | | TCGA(*N*=374) | | GEO(*N*=81) | |
| --- | --- | --- | --- | --- | --- |
|  |  | NO | % | NO | % |
| Sex | Female | 122 | 32.62% | 17 | 20.99% |
|  | Male | 252 | 67.38% | 64 | 79.01% |
| Age |  | 61.00 [51.00;69.00] | | - | |
| Pathologigal  stage | I | 175 | 46.79% | 16 | 19.75% |
|  | II | 87 | 23.26% | 22 | 27.16% |
|  | III | 86 | 22.99% | 31 | 38.27% |
|  | IV | 5 | 1.34% | 5 | 6.17% |
|  | Unkown | 21 | 5.62% | 7 | 8.65% |
| T | T1 | 185 | 49.47% | - | - |
|  | T2 | 95 | 25.40% |  |  |
|  | T3 | 81 | 21.66% |  |  |
|  | T4 | 12 | 3.21% |  |  |
|  | unknow | 1 | 0.26% |  |  |
| N | N0 | 257 | 68.72% | - | - |
|  | N1 | 4 | 1.07% |  |  |
|  | NX | 112 | 29.94% |  |  |
|  | unknow | 1 | 0.27% |  |  |
| M | M0 | 272 | 72.73% | - | - |
|  | M1 | 4 | 1.07% |  |  |
|  | MX | 98 | 26.20% |  |  |
| Survival time |  | 598.50 [329.50;1088.75] | | 731.85±384.82 | |
| OS status | 0 | 242 | 64.71% | 16 | 19.8% |
|  | 1 | 132 | 35.29% | 65 | 80.2% |
