# Supplementary material for: A prognostic model based on DNA methylation-related gene expression for predicting overall survival in hepatocellular carcinoma
Source: Front Oncol. 2024 Jan 18;13:1171932. doi: 10.3389/fonc.2023.1171932 (PMC10830715; doi:10.3389/fonc.2023.1171932)
Supplement: Supplementary file 3 [file Table_3.docx]

**Table S3**

| Characteristics | | TCGA(*N*=374) | | |
| --- | --- | --- | --- | --- |
|  |  | NO | % | |
| Age |  | 1 | |  |
| Stage | Unkown | 21 |  | |
| Grade | unknow | 1 |  | |
| Survival time <30 days |  | 20 |  | |
