# Supplementary figures and images for: A prognostic model based on DNA methylation-related gene expression for predicting overall survival in hepatocellular carcinoma
Source: Front Oncol. 2024 Jan 18;13:1171932. doi: 10.3389/fonc.2023.1171932 (PMC10830715; doi:10.3389/fonc.2023.1171932)

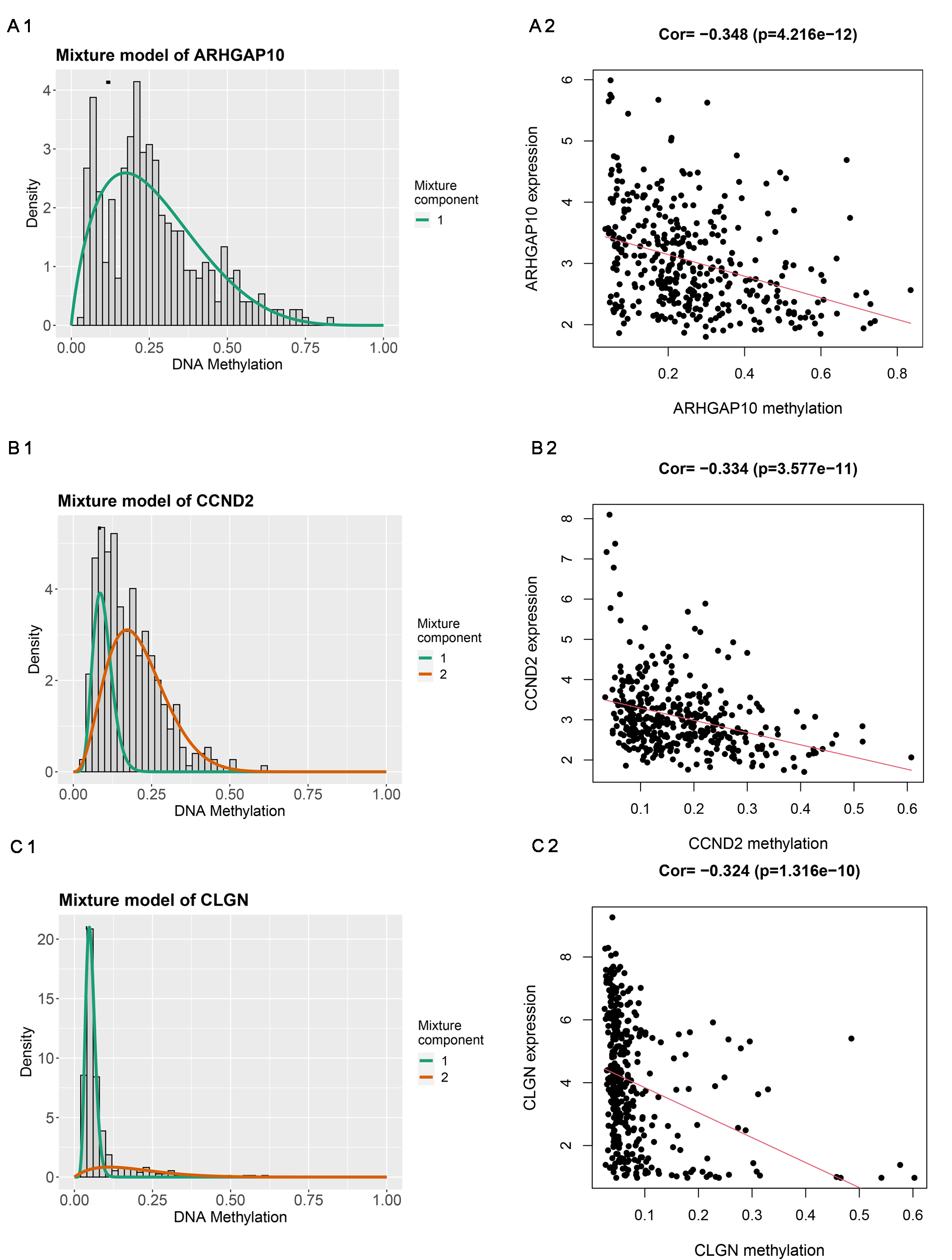

Supplement: Supplementary Figure 1 — Several of the 17 DNA methylation-driven genes. [file Image_1.png]

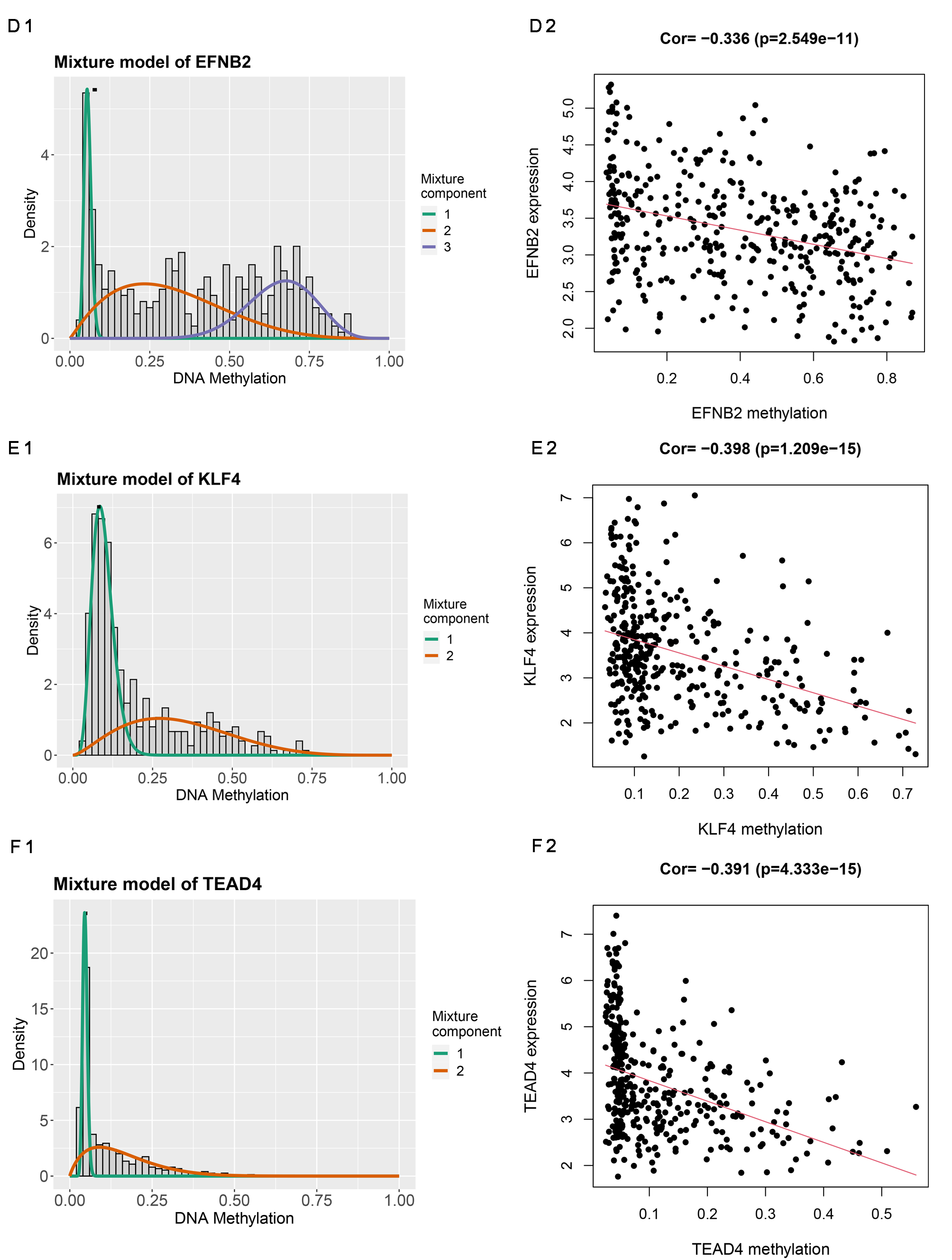

Supplement: Supplementary Figure 2 — 10 DNA methylation-driven geneswere identified as being associated with prognosis. [file Image_2.png]

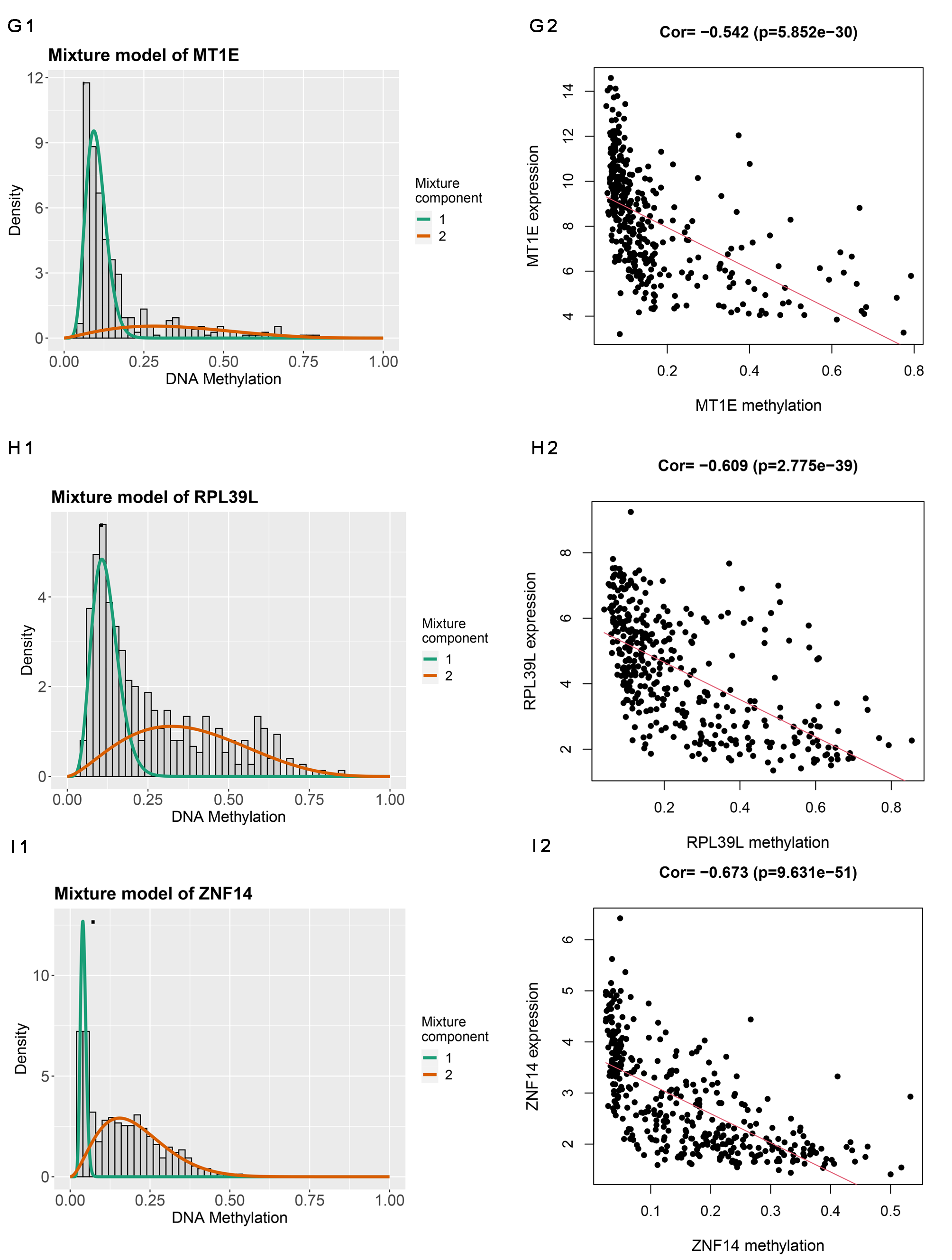

Supplement: Supplementary Figure 3 — Building the combined nomogram to predict the overall survival (OS) of patients with HCC. [file Image_3.png]

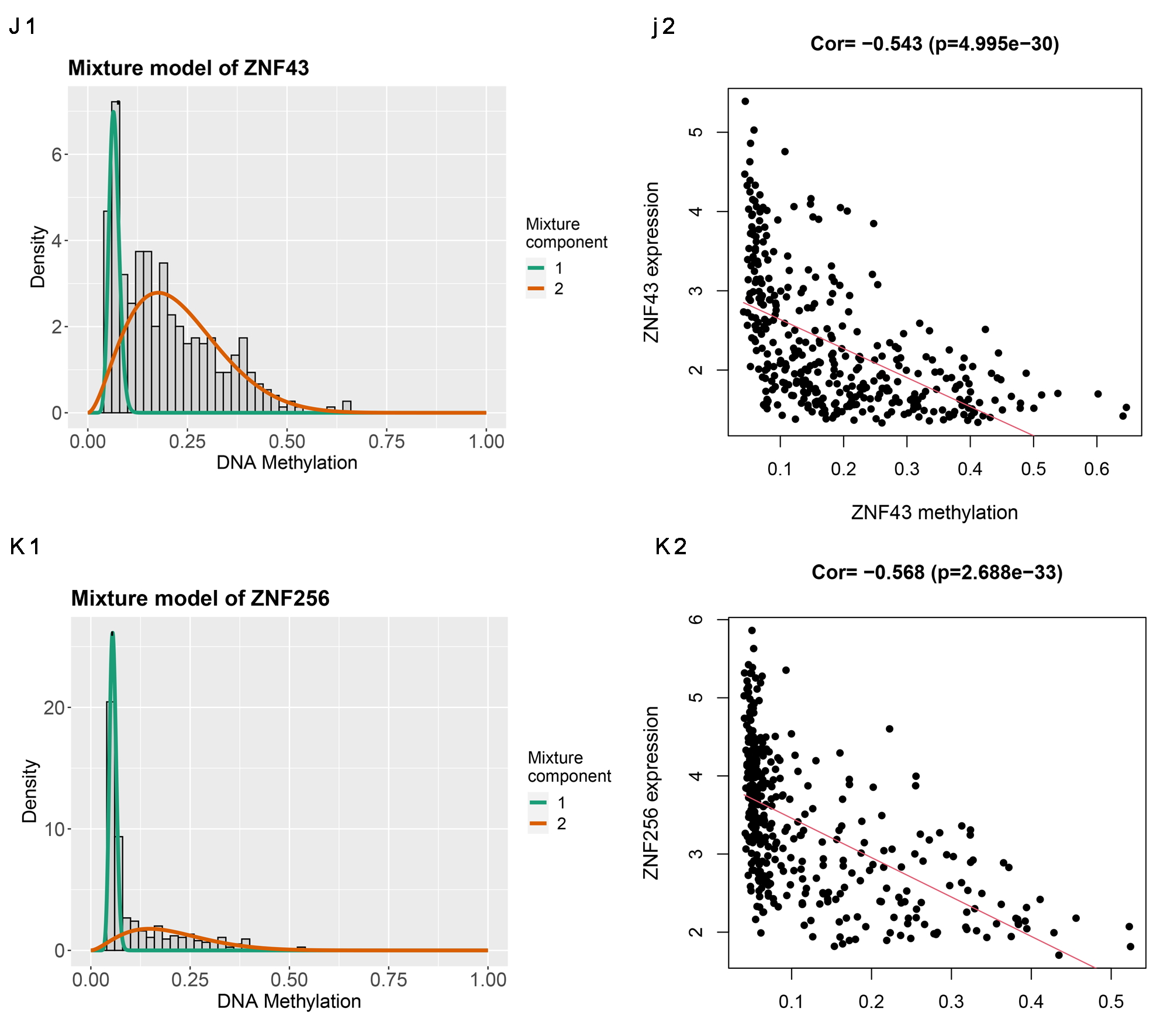

Supplement: Supplementary file 8 [file Image_4.png]

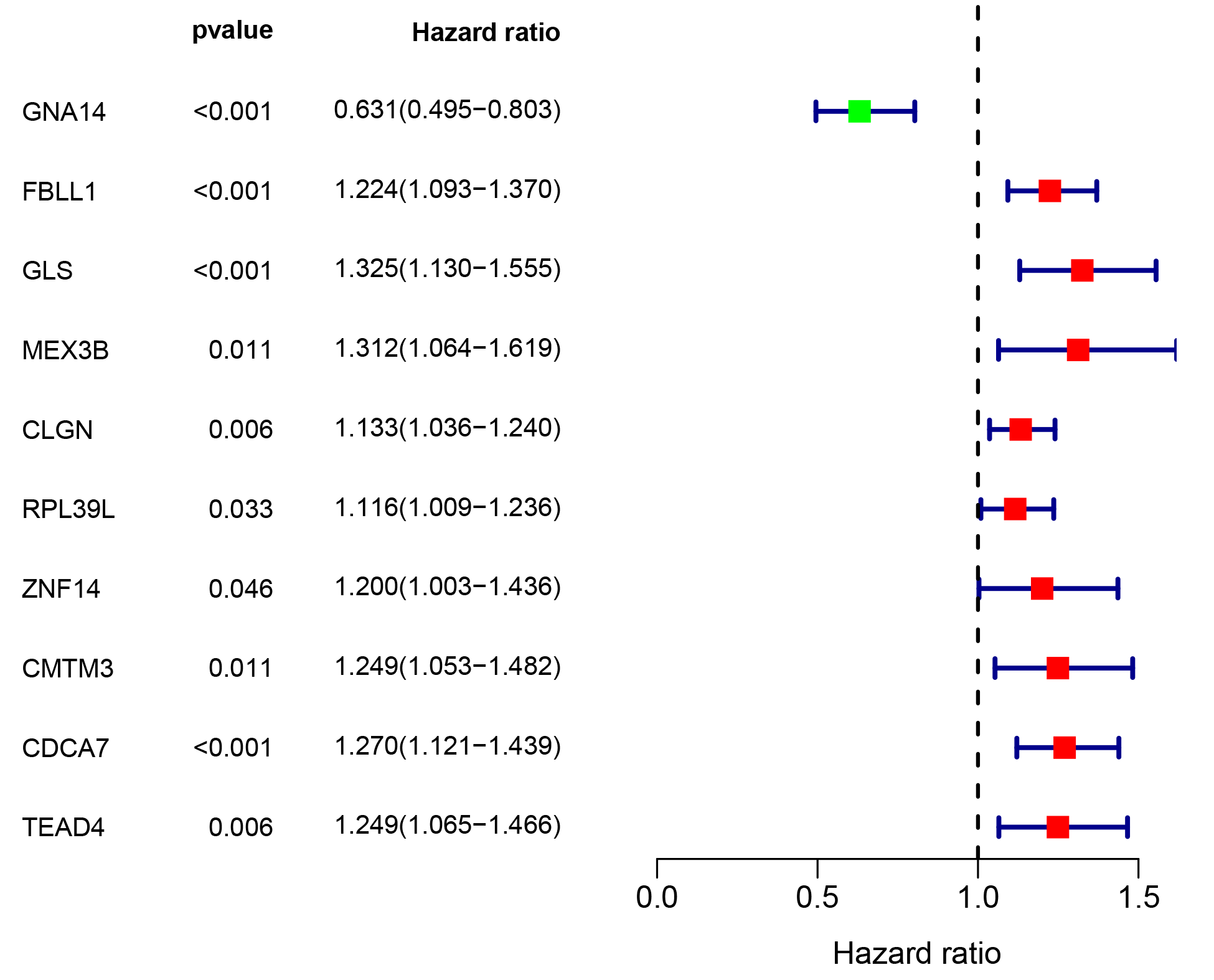

Supplement: Supplementary file 9 [file Image_5.png]

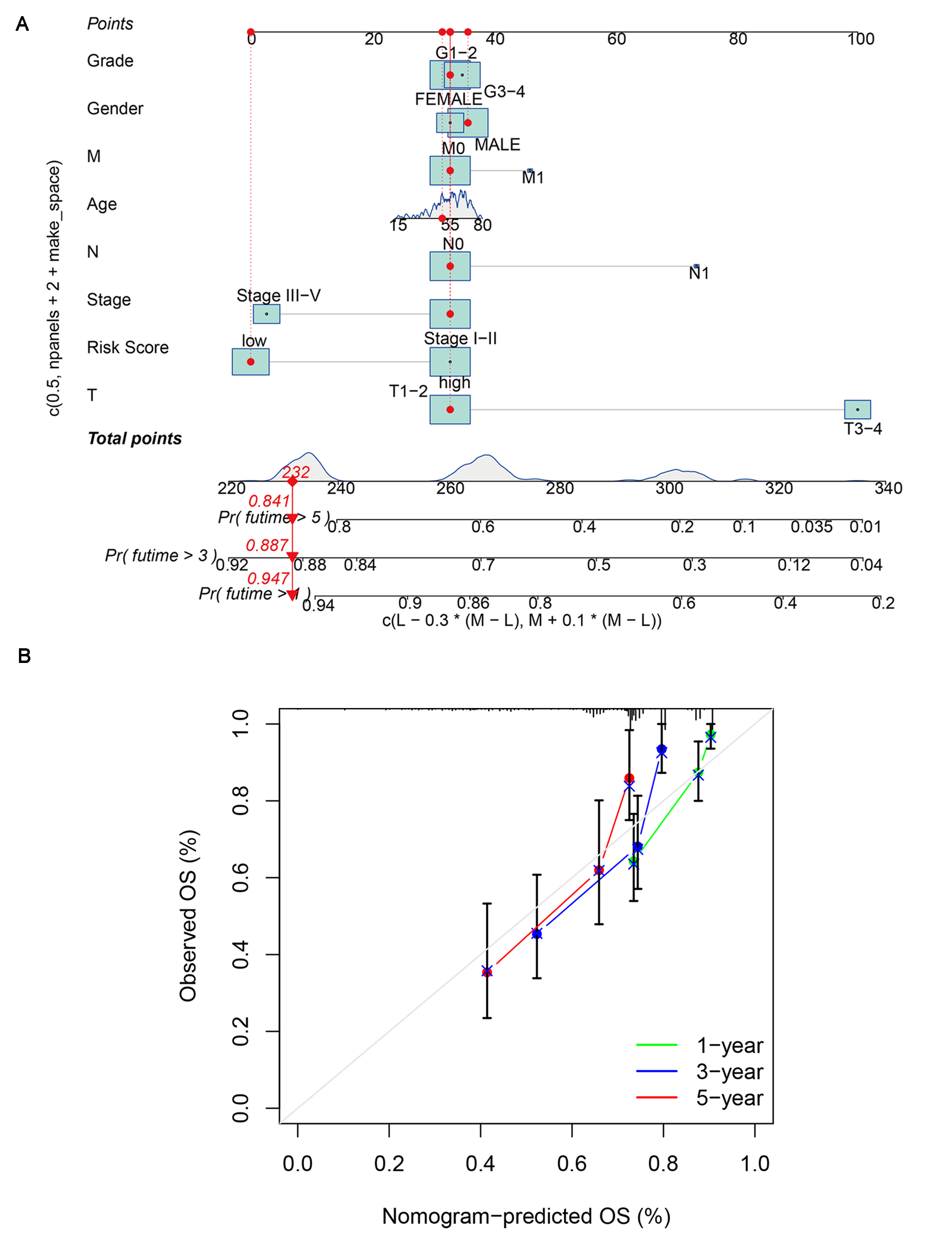

Supplement: Supplementary file 10 [file Image_6.png]
